# 

**Published:** 1860-04

**Authors:** 


### RECEIPTS FOR THE JOURNAL FOR MARCH, 1860.

| Name | Vol. | $ | Name | Vol. | $ |
|---|---|---|---|---|---|
| H. Carpenter, Iowa | Vol. 3 | $2 | L. D. Smeadley, Ill | Vol. 3 | $2 |
| Thomas A. Brown, Ill. | " 3 | 2 | J. W. York, Ill | " 3 | 2 |
| Milton Parker, Ill. | " 2 | 2 | H. R. Payne, Ill | " 1-2 | 4 |
| W. C. Brown, Wis | " 3 | 2 | Thos. A. Graham, Ind | " 3 | 2 |
| R. Willson, Wis | " 1-2 | 4 | H. Warne, Wis | " 3 | 2 |
| A. W. Adair, Ind | " 3 | 2 | Jas. H. Farris, Ill | " 3 | 2 |
| James P. Tucker, Ind | " 3 | 2 | C. Goodbrake, Ill | " 3 | 1 |
| Benj. P. Estes, Ky | " 3 | 2 | S. B. Bennett, Ill | " 3 | 2 |
| James C. McCoy, Ill | " 1-2-3 | 6 | D. S. Jenks, Ill | " 3 | 2 |
| E. L. Holmes, Ill | " 3 | 2 | C. D. Henton, Ill | " 3 | 2 |
| G. W. Noble, Ill | " 2-3 | 4 | Wm. G. Elder, Mo | " 3 | 2 |
| O. Allen, Min | " 1 | 2 | R. Q. Wilson, Ind | " 3 | 2 |
| J. W. Cowden, Iowa | " 3 | 2 | C. J. M. Van Hosen, Ill | " 3 | 2 |
| Robert W. Earll, Wis | " 3 | 2 | L. B. Brown, Ill | " 3 | 2 |
| Ira E. Oatman, Cal | " 3 | 2 | D. W. Hand, Min, to No. 8 | " 3 | 2 |
| G. S. Barrow, Ill | " 3 | 2 | J. T. Pearman, Ill | " 3 | 2 |

☞ The American Medical Association will hold its Thirteenth Annual Meeting at New Haven, on the first Tuesday of June, 1860. The Secretaries of Local Societies, Colleges, and Hospitals, are requested to forward the names of delegates as soon as they are appointed, to

Stephen G. Hubbard, M. D., Secretary,
New Haven, Ct.

